# Timing of treatment in osteosarcoma: challenges and perspectives – a scoping review

**DOI:** 10.1186/s12885-022-10061-0

**Published:** 2022-09-10

**Authors:** Michael S. Kim, Ioanna K. Bolia, Brenda Iglesias, Tamara Sharf, Sidney I. Roberts, Hyunwoo Kang, Alexander B. Christ, Lawrence R. Menendez

**Affiliations:** 1 Department of Orthopaedic Surgery, Keck School of Medicine of USC, 1520 San Pablo St, HC2 #2000, Los Angeles, CA 90033 USA; 2 Department of Orthopaedic Surgery, University of Pittsburgh School of Medicine, Pittsburgh, PA USA

**Keywords:** Osteosarcoma, Orthopaedic surgery, Timing, Oncology, Surgery, Chemotherapy, Radiation

## Abstract

**Background:**

The timing of events in the management of osteosarcoma may be critical for patient survivorship; however, the prognostic value of factors such as onset of symptoms or initiation of therapy in these patients has not been studied. This study sought to review the literature reporting treatment of osteosarcoma to determine the utility of event timing as a prognostic indicator. Due to significant heterogeneity in the literature, this study was conducted as a scoping review to assess the current state of the literature, identify strengths and weaknesses in current reporting practices, and to propose avenues for future improvement.

**Main body:**

This review screened 312 peer-reviewed studies of osteosarcoma in any anatomic location published in an English journal for reporting of an event timing metric of any kind in a population of 6 or more. Thirty-seven studies met inclusion/exclusion criteria and were assessed for level of evidence, quality, and event timing metric. Reviewers also collated: publication year, population size, population age, tumor site, tumor type, surgical treatment, and adjuvant medical treatment. Extracted event timing data were further characterized using nine standardized categories to enable systematic analysis. The reporting of event timing in the treatment of osteosarcoma was incomplete and heterogenous. Only 37 of 312 (11.9%) screened studies reported event timing in any capacity. The period between patient-reported symptom initiation and definitive diagnosis was the most reported (17/37, 45.9%). Symptom duration was the second most reported period (10/37, 27.0%). Event timing was typically reported incidentally and was never rigorously incorporated into data analysis or discussion. No studies considered the impact of event timing on a primary outcome. The six largest studies were assessed in detail to identify pearls for future researchers. Notable shortcomings included the inadequate reporting of the definition of an event timing period and the pooling of patients into poorly defined timing groups.

**Conclusions:**

Inconsistent reporting of event timing in osteosarcoma treatment prevents the development of clinically useful conclusions despite evidence to suggest event timing is a useful prognostic indicator. Consensus guidelines are necessary to improve uniformity and utility in the reporting of event timing.

**Supplementary Information:**

The online version contains supplementary material available at 10.1186/s12885-022-10061-0.

## Background

Osteosarcoma is a rare bone malignancy with a global incidence under 30,000 individuals per year [1, 2]. Adjuvant chemotherapy has increased five-year survival rates three-fold to over 65%, and surgical advances have made radical resection with limb salvage possible in greater than 90% of cases [3]. Despite the marked improvement in outcomes, low case counts make it challenging to recruit sufficient populations to power high-quality studies. Meta-analysis of the studies that have been conducted is also challenging because of highly variable reporting of specific event timing periods [4–8].

Event timing metrics such as the period between symptom presentation and definitive diagnosis or between patient presentation and the initiation of medical treatment are important to compare studies and outcomes and to assess the utility of an event timing period as a prognostic indicator. Accurately quantifying the time from symptom onset or diagnosis to the initiation of treatment is of particular interest given the association of treatment delay with poorer outcomes [4, 9]. There is no consensus on what event timing metrics are critical and how they should be reported.

This scoping review’s primary objective was to describe the reporting of time to the initiation of treatment in osteosarcoma with a focus on statistics quantifying the period prior to presentation and the initiation of therapy. Challenges and limitations identified in the process of conducting this scoping review have elucidated the need for consensus guidelines for event timing metrics to enable the comparison of results across studies, and ultimately, to improve outcomes.

## Methods

**Fig. 1:**
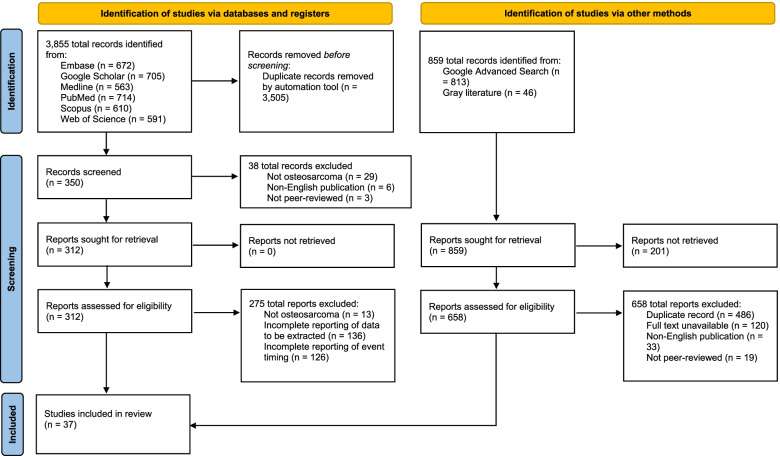
PRISMA flow diagram for new systematic reviews which included searches of databases, registers, and other sources

This study was conducted as a scoping review per Preferred Reporting Items for Systematic Reviews and Meta-Analyses – Scoping Review (PRISMA-ScR) guidelines due to significant heterogeneity among studies reporting event timing in the treatment of osteosarcoma [10]. The Embase, Google Scholar, Medline, PubMed, Scopus, and Web of Science databases, Google Advanced Search, and gray literature were queried for studies reporting event timing in the treatment of osteosarcoma from inception to September 10, 2021. The following search terms were combined by “AND” or “OR” to retrieve potentially eligible articles: “osteosarcoma,” “outcomes,” “timing,” and anatomic site (e.g., “pelvis,” “lower extremity”). A full list of search terms is provided as Additional file 1. Inclusion criteria were (1) full text publication in English in a peer-reviewed journal, (2) prospective or retrospective primary studies, (3) population equal to or greater than 6 patients, (4) treatment for osteosarcoma, (5) reported time to treatment, (6) complete reporting of data to be extracted. Extracted data included: publication year, level of evidence, population size, population age, tumor site, tumor type, surgical treatment, adjuvant medical treatment, and event timing. Data was compiled in an Excel spreadsheet (Microsoft, Redmond, CA). Overall averages (e.g., symptom duration) were calculated by weighting reported means by study population without regard for level of evidence or study design beyond this study’s inclusion criteria. The reference list of included studies was searched manually for further potentially eligible articles. Two reviewers screened each potentially eligible study by title, abstract, and full text, extracted data, and assessed study quality using the Mixed Methods Appraisal Tool (MMAT) [11]. Time to treatment statistics were classified by nine categories. Discrepancies between reviewers were resolved by a third reviewer. The study identification and screening process is presented in Fig. 1.

## Results

The initial literature search yielded 312 studies of which 37 met the inclusion criteria. Inter-rater agreement among two reviewers was high in title and abstract screening (90% agreement), full text screening (90% agreement), and study quality assessment (95% agreement). As demonstrated in Fig. 1, general search engines (e.g., Google Advanced Search) were queried in addition to academic databases or registers. This resulted in 859 records identified. However, none were ultimately included in this review. The majority of records identified through general search engines were duplicates of records identified through academic databases. The 201 reports not retrieved occurred due to a promising search result, which was included in the 859 total records identified, led to an inactive website that continued to be indexed by Google or a report eligible for this study was not available on the website.

In total, 4594 patients (2672 males, 1922 females) were included in this study. Five studies (5/37, 13.5%) accounting for 772 patients (772/4814, 16.0%) did not report age in a manner that could be meta-analyzed. The mean patient age of 4042 patients (4042/4814, 84.0%) was 17.1 years (range 0–90 years). The median population was 40 patients with a range of seven to 2442 patients. Only six studies (6/37, 16.2%) included populations equal to or greater than 121 patients [4–7, 12, 13]. Per American Academy of Orthopedic Surgeons criteria, 20 (20/37, 54.1%) studies were of Level of Evidence IV, 14 studies were Level III (14/37, 37.8%), and 3 studies were Level II (3/37, 8.1%) [14]. Included studies are summarized in Table 1 with additional extracted data available as Additional file 2. Per MMAT criteria, 27 studies (27/37, 73.0%) were of high quality, meeting 100% of the tool’s five criteria. Seven studies (7/37, 18.9%) met 80%. Two studies (2/37, 5.4%) met 60%. One study (1/37, 2.7%) met 40%. MMAT grading is reported fully in Additional file 3.

**Table 1:** Summary of population characteristics and event timing category of included studies

Study	Level of Evidence	Total Population	Male	Female	Mean Age	Minimum Age	Maximum Age	Category
Amr et al., 2000	III	23	11	12	18.6	6	45	8
Bacci et al., 2009	III	55	34	21	17.9	3	40	8
Bertoni et al., 2005	III	29	12	17	36	15	65	8
Chow et al., 2000	III	14	7	7	Not reported	7	21	8
Daecke et al., 2005	III	39	18	21	Not reported	5	59	2
Daugaard et al., 1987	III	87	60	27	Not reported	Not reported	Not reported	6
Donati et al., 2004	III	60	30	30	Not reported	8	66	8
Evans et al., 2020	III	2442	1472	970	18	13	32	6
Feng et al., 2013	III	16	10	6	37.1	15	58	2
Fuchs et al., 1998	II	171	107	64	Not reported	Not reported	Not reported	6, 7
Fuchs et al., 2009	II	43	29	14	34.4	11	66	2, 8
Guo et al., 1981	IV	12	9	3	Not reported	13	60	9
Ham et al., 2000	II	40	19	21	Not reported	13	83	2, 8
Hu et al., 2010	III	18	12	6	48	34	65	2
Jamshidi et al., 2017	IV	7	4	3	25.1	7	49	8
Kager et al., 2010	III	28	16	12	Not reported	2.2	4.9	2
Kozlowski et al., 1988	IV	21	11	10	11.4	0.5	18	9
Lawrence et al., 1993	IV	47	23	24	12	4	20	7
Letaief et al., 2020	III	85	53	32	17	1	62	1, 6
Makley et al., 1988	III	166	85	81	Not reported	Not reported	Not reported	7
Ozaki et al., 2002	IV	22	9	13	Not reported	5	55	2
Ozaki et al., 2003	IV	67	41	26	Not reported	10	63	2
Parry et al., 2016	IV	121	47	74	29.3	9	76	2
Pylkkanen et al., 1997	IV	36	21	15	28	5	62	2
Rao et al., 1978	IV	8	4	4	58.7	Not reported	Not reported	2
Saeter et al., 1991	IV	97	64	33	Not reported	6	36	2
Sathiyamoorthy and Ali, 2012	IV	20	11	9	17.1	5	48	2
Sordillo et al., 1983	IV	48	23	25	Not reported	6	80	2
Stein, 1975	IV	46	26	20	26.7	5	73	2
Taylor et al., 1985	IV	336	188	148	Not reported	Not reported	Not reported	2
Thomas et al., 2014	IV	7	5	2	36	15	54	7
Trieb et al., 2013	III	49	28	21	21.8	9	53	6
Tsagozis et al., 2019	IV	256	147	109	20	0	90	2
VandenBuscche et al., 2016	IV	17	9	8	29.2	12	70	2
Zileli et al., 2003	IV	34	14	20	42	14	71	2
Zils et al., 2013	IV	20	11	9	31	5	58	2
Zils et al., 2015	IV	7	2	5	13	7	16	2

### Event timing categories

Nine event timing periods were defined during the study screening process based on the authors’ identification of common trends in event timing reporting. Thirty-three studies (33/37, 89.2%) reported event timing using one defined period while five studies (4/37, 10.8%) reported event timing using two defined periods for a total of 41 defined event timing periods reported in 37 studies. The number of times each period was used to report event timing is summarized in Table 2.

**Table 2:** Summary of number of studies by event timing category

Period	Start	End	Reported in Studies
1	Patient-reported symptom initiation	Initial presentation	5
2	Patient-reported symptom initiation	Definitive diagnosis	17
3	Patient-reported symptom initiation	Medical therapy initiation	0
4	Patient-reported symptom initiation	Surgical intervention	2
5	Initial presentation	Definitive diagnosis	0
6	Initial presentation	Medical therapy initiation	2
7	Initial presentation	Surgical intervention	0
8	Symptom duration		10
9	Other		5

### Period between patient-reported symptom initiation and initial presentation

Five studies (5/37, 13.5%) including 259 patients (259/4594, 5.6%) reported this period [9, 15–18]. The average period between symptom initiation and initial presentation was 4.4 months with a range of 0 to 48 months. Pain was the most reported symptom reported by 87 patients (87/259, 33.6%). A palpable mass was reported by 44 patients (44/259, 17.0%) and peripheral nerve involvement was reported by 27 patients (27/259, 10.4%). No initiating symptom was specified for 101 patients (101/259, 39.0%).

### Period between patient-reported symptom initiation and definitive diagnosis

Seventeen studies (17/37, 45.9%) including 907 patients (907/4594, 19.7%) reported this period [4, 5, 19–33]. The average period between symptom initiation and definitive diagnosis was 1.39 months with a range of 0 to 24 months based on 9 studies (9/17, 52.9%) including 302 patients (302/907, 33.3%) that reported a mean period [20, 23–27, 29–31]. Eight studies (8/17, 47.1%) did not report this period in a manner that could be incorporated in the overall mean. Five studies (5/17, 29.4%) including 110 patients (110/907, 12.1%) reported symptoms on an individual basis [19, 21–23, 28]. Of these patients, 61 (61/110, 55.5%) reported pain, 11 (11/110, 10.0%) reported swelling, nine (9/110, 8.2%) reported neurologic dysfunction, and 3 reported pathologic fracture (3/110, 2.7%). The initiating symptom was not reported for the remaining 26 patients (26/110, 23.6%).

### Period between patient-reported symptom initiation and medical therapy initiation

No studies reported this period.

### Period between patient-reported symptom initiation and surgical intervention

Two studies (2/37, 5.4%) including 200 patients (200/4594, 4.4%) reported this period [6, 34]. Data was reported inconsistently. One study (166/200, 83.0%) reporting this period as 0–1 month (*n* = 37), 2 months (*n* = 39), and 3 months or more (*n* = 55) [6]. The other study (34/200, 17.0%) reported a mean period of 20 months with a range of 7 days to 10 years [34].

### Period between initial presentation and definitive diagnosis

No studies reported this period.

### Period between initial presentation and medical therapy initiation

Two studies (2/37, 5.4%) including 256 patients (256/4594, 5.6%) reported this period [9, 12]. One study (171/256, 66.8%) reported this period incidentally by only enrolling patients who started treatment within three weeks of initial presentation [12]. The other study (85/256, 33.2%) reported a mean period of 27 days with a range of 3 to 85 days [9].

### Period between initial presentation and surgical intervention

No studies reported this period.

### Symptom duration

Ten studies (10/37, 27.0%) including 559 patients (559/4594, 12.2%) reported this period [13, 31–33, 35–40]. The average period of symptom duration was 9.9 months with a range of 0 to 36 months based on nine studies (9/10, 90.0%) including 536 patients (536/559, 95.9%) that reported a mean period [13, 31–33, 36–40].

### Other

Five studies (5/37, 13.5%) including 2566 patients (2566/4594, 55.9%) reported a statistic quantifying time to treatment other than the categories previously listed [7, 8, 41–43]. Two studies including 28 patients (28/2566, 1.1%) reported event timing on an individual basis and in a manner that could not be meta-analyzed [8, 42]. Three other studies including 2538 patients (2538/2566, 98.9%) reported the period between definitive diagnosis and medical therapy initiation and/or surgical intervention [7, 41, 43]. The average period between diagnosis and medical therapy was 21.0 days with a range of 0 to 24.5 days based on two studies (2/5, 40.0%) including 2491 patients (2491/2566, 97.1%) [7, 43]. The average period between diagnosis and surgical intervention was 93.0 days with a range of 35.0 to 98.1 days based on three studies (3/5, 60.0%) including 2566 patients (2566/2566, 100.0%) [7, 41].

## Discussion

This scoping review classified event timing statistics as reported in 37 studies by nine categories to identify trends in the reporting of time prior to the initiation of treatment of osteosarcoma. Most screened studies did not report event timing statistics in any form demonstrating the need for consensus guidelines in reporting. The most reported period was the time between patient-reported symptom initiation and definitive diagnosis (45.9%). The second most reported period was the time between patient-reported symptom initiation and initial presentation (13.5%). Tied for the third most reported periods were the time between patient-reported symptom initiation and surgical intervention and the time between initial presentation and the initiation of medical therapy (5.4%). The time between patient-reported symptom initiation and the initiation of medical therapy, the time between initial presentation and definitive diagnosis, and the time between initial presentation and surgical intervention were not reported in any studies. Overall symptom duration was commonly reported as a measure of time to treatment despite the poor and variable definition of its start and end points (27.0%). Other statistics that were not defined prior to this study (i.e., the time between definitive diagnosis and the initiation of medical treatment and the time between definitive diagnosis and surgical intervention) were tied for the second most reported periods (13.5%).

No studies assessed the association of patient-reported initiation of symptoms to definitive diagnosis (Category 2) with outcomes. A study of 121 patients suffering from pelvic osteosarcoma presenting to a single institution over 31 years found that metastases at the time of diagnosis had a significant impact on five-year survival rates, but did not consider whether delay in diagnosis impacted the rate of metastases found at diagnosis [4]. A study of 256 patients suffering from chondroblastic osteosarcoma (COS) who presented to a single institution over 37 years reported a mean duration of symptoms of 16 weeks with an interquartile range of six to 18 (Category 8) [13]. While reporting a high incidence of metastases in COS patients, this study did not consider whether time before definitive diagnosis was a contributing factor to the incidence of metastases at the time of diagnosis and whether either statistic had an impact on prognosis. Despite the intuitive connection between delay and presence of metastases and what metastases mean for prognosis, it is impossible to make conclusions about either event timing categories’ utility as a prognostic tool. Future studies of osteosarcoma treatment should include event timing periods in their assessment of end points.

Many studies reported timing in a manner that was unclear and impossible to incorporate in meta-analysis. A study of 336 patients suffering from osteosarcoma in the extremities presenting to a single institution over 18 years reported the period from patient-reported initiation of symptoms to definitive diagnosis (Category 2) using ranges that prevented meta-analysis: 145 patients presented after 1–2 months of symptoms, 78 patients presented after 3–5 months of symptoms, and 98 patients reported after 6 or more months of symptoms [5]. No statement was made as to whether the reported boundaries are inclusive or exclusive. Another large study reported on 166 children with osteosarcoma of the extremities who presented to the Childrens Cancer Study Group institutions over 15 years and exhibited the same inadequate reporting of event timing (Category 4) using poorly defined ranges [6]. Consensus guidelines would facilitate the reporting of event timing data in a manner that is useful to current readers and to future researchers conducting meta-analysis.

The largest study in this review included 2442 patients suffering from osteosarcoma in the extremities in the National Cancer Database study and reported time from definitive diagnosis to surgery, a period not reported by any other study included in this review (Category 9) [7]. While this is an interesting metric, the time from diagnosis to surgery is most likely a reflection of an institution’s operational capacity and the physicians’ decision whether to perform initial surgery versus treat with neoadjuvant chemotherapy, rather than a useful contributor to prognosis.

None of the included studies used an event timing period as an outcome, nor did they discuss the contribution of event timing periods to prognosis. In every study included in this review, event timing was reported alongside demographic data as a characterization of the population, but not a prognostic contributor to outcomes. The remarkable improvement in outcomes among patients diagnosed with osteosarcoma has largely been attributed to the widespread adoption of adjuvant chemotherapy and improved surgical technique. As the treatment of osteosarcoma is standardized, the potential contribution of event timing to prognosis and its utility as an adjunct to tumor staging in the development of patient-specific treatment plans is critical. Due to the complete absence of consideration of the impact of event timing on prognosis and outcomes in the studies included in this review, it is impossible to draw conclusions about the effect of duration of time to diagnosis and treatment on clinical outcomes and survival rates in patients suffering from osteosarcoma. Intuition suggests that delays in treatment allow tumor progression and the development of metastases, which are known to have a negative impact on outcomes and survival. This review demonstrates the need to further investigate the impact of event timing metrics, particularly those quantifying the periods preceding presentation and the initiation of treatment, on functional outcomes and survival. The development of expert consensus statements, such as those produced based on Delphi questionnaires, [44, 45] may be useful in identifying and defining critical event timing metrics and producing reporting guidelines.

### Limitations

This study has some limitations. While conducted systematically, this review may have missed eligible studies. It is possible that some authors did not report event timing data that had been collected in detail due to limitations imposed by the publication process, particularly as event timing was not a primary outcome measure in any included study. Ideally, the literature would have reported event timing in a manner that allowed for the identification of periods critical to treatment optimization and improving outcomes. However, this review was significantly limited by the discrepancies in the reporting of event timing, which resulted in very few of the screened studies being included and the inability to utilize coherent categories of event timing statistics that enabled meta-analysis. Due to the limited number of studies and small populations, this study had expansive inclusion criteria and pooled patients with osteosarcoma regardless of demographics, tumor site, and treatment. This prevented the association of event timing with clinical outcomes in this study. The results of this scoping review elucidate the need for further study of event timing and the development of consensus guidelines in the reporting of event timing to facilitate improvements in the treatment of osteosarcoma.

## Conclusions

Event timing in the treatment of osteosarcoma may have an impact on prognosis, outcomes, and survival. However, event timing metrics are currently reported inconsistently and have not been studied rigorously. As a result, it is currently impossible to draw conclusions as to their utility as prognostic markers and further research is required to elucidate their use as a clinical tool and consensus guidelines to make reporting uniform and meta-analyzable should be considered.

## Supplementary Information

**Additional file 1.**

**Additional file 2.**

**Additional file 3.**

## Data Availability

The datasets used and/or analysed during the current study are available from the corresponding author on reasonable request and have been provided to the journal as supplementary material.

## Abbreviations

PRISMA-ScR: Preferred Reporting Items for Systematic Reviews and Meta-Analyses – Scoping Review
MMAT: Mixed Methods Appraisal Tool
