# Supplementary material for: Timing of treatment in osteosarcoma: challenges and perspectives – a scoping review
Source: BMC Cancer. 2022 Sep 10;22:970. doi: 10.1186/s12885-022-10061-0 (PMC9464396; doi:10.1186/s12885-022-10061-0)
Supplement: Supplementary file 1 — Additional file 1. [file 12885_2022_10061_MOESM1_ESM.docx]

***Appendix A.*** *Search Terms.*

- “osteosarcoma” AND “timing” AND “upper extremity”
- “osteosarcoma” AND “timing” AND “lower extremity”
- “osteosarcoma” AND “timing” AND “spine”
- “osteosarcoma” AND “timing” AND “pelvis”
- “osteosarcoma” AND “outcome” AND “upper extremity”
- “osteosarcoma” AND “outcome” AND “lower extremity”
- “osteosarcoma” AND “outcome” AND “spine”
- “osteosarcoma” AND “outcome” AND “pelvis”
